# Association of hypoalbuminemia and reversal of albumin-to-globulin ratio with morbidity outcome among hospitalized Lassa fever infected patients at a dedicated treatment center in Ondo state, south-western Nigeria

**DOI:** 10.2144/fsoa-2020-0075

**Published:** 2020-08-13

**Authors:** Sampson Omagbemi Owhin, Chukwuyem Abejegah, Olumuyiwa John Fasipe, Clement Oke, Abiodun Abidoye, Austine Osagbaekhoe, Abimbola Awe, Ijeoma Etafo, Evbaruese Iredia, Olufemi Ayodeji, Lanre Olatunde, Olalekan Ojo, Josephine Alabi, Peter Ehizokhale Akhideno, Harrison Ejiyere, Azuka Stephen Adeke, Joachim Azegbeobor, Liasu Ahmed

**Affiliations:** 1 Department of Internal Medicine and Hemato-oncology Unit, Federal Medical Center Owo, Ondo State, Nigeria; 2 Department of Community Medicine, Federal Medical Center Owo, Ondo State, Nigeria; 3 Department of Clinical Pharmacology and Therapeutics, Faculty of Basic Clinical Sciences, University of Medical Sciences, Ondo City, Ondo State, Nigeria; 4 Department of Nursing, Federal Medical Center Owo, Ondo State, Nigeria; 5 Department of Internal Medicine, Irrua Specialist Teaching Hospital Irrua, Edo State, Nigeria; 6 Department of Community Medicine, Alex Ekwueme Federal University Teaching Hospital Abakaliki, Ebonyi State, Nigeria; 7 Department of Psychiatry, Federal Medical Center Owo, Ondo State, Nigeria; 8 Office of the Chief Medical Director, Federal Medical Center, Owo, Ondo State, Nigeria

**Keywords:** Federal Medical Center Owo, hospitalized, hypoalbuminemia, Lassa fever infection, morbidity outcome, Nigeria, reversal of albumin-to-globulin ratio

## Abstract

**Background:**

As of this present moment, there is paucity of data on report concerning the association between hypoalbuminaemia or reversal of albumin-to-globulin ratio and morbidity outcome in Lassa fever (LF) infection as a crucial determinant prognostic-predictor factor for treatment-survival outcome.

**Aim:**

This study was designed to determine the association between hypoalbuminaemia, reversal of albumin-to-globulin ratio and morbidity outcome among confirmed LF infected patients.

**Methodology:**

This was a descriptive retrospective study involving the assessment of records of confirmed LF infected patients that were managed at the center from November 2018 to October 2019.

**Results:**

Out of 83 recruited participants with complete records, 66 (79.5%) had hypoalbuminaemia, 74 (89.2%) had reversal of albumin-to-globulin ratio. A higher mean value of total white blood cell (WBC) count was observed among patients with hypoalbuminaemia (p < 0.0001) and reversal of albumin-to-globulin ratio (p < 0.0001) when compared to patients with normal values, respectively. Also, this study showed statistically significant associations between serum albumin level versus total WBC count (p < 0.0001), acute kidney injury (AKI; p = 0.009), bleeding diathesis (p < 0.0001), and occurrence of pregnancy miscarriage (p < 0.0001).

**Conclusion:**

There is a baseline hypoalbuminaemia and reversal of albumin-to-globulin ratio among confirmed LF infected patients. Based on these findings, the serum level of albumin and albumin-to-globulin ratio at presentation may serve as simple early biomarkers to identify patients at high risk for a complicated clinical course of disease. This study also reveals that those hospitalized LF infected patients with hypoalbuminemia and/or reversal of albumin-to-globulin ratio tend to have leucocytosis and experience prolonged duration of illness.

## Lay abstract

This study was designed to determine the association between hypoalbuminaemia, reversal of albumin-to-globulin ratio and morbidity outcome among confirmed LF infected patients from November 2018 to October 2019. Out of 83 recruited participants with complete records; 66 (79.5%) had hypoalbuminaemia, 74 (89.2%) had reversal of albumin-to-globulin ratio. A higher mean value of total white blood cell (WBC) count was observed among patients with hypoalbuminaemia (p < 0.0001) and reversal of albumin-to-globulin ratio (p < 0.0001) when compared to patients with normal values, respectively. Also, this study showed statistically significant associations between serum albumin level versus total WBC count (p < 0.0001), acute kidney injury (AKI; p = 0.009), bleeding diathesis (p < 0.0001), and occurrence of pregnancy miscarriage (p < 0.0001). There is a baseline hypoalbuminaemia and reversal of albumin-to-globulin ratio among confirmed LF infected patients. Based on these findings, the level of albumin and albumin-to-globulin ratio at presentation may serve as simple early biomarkers to identify patients at high risk for a complicated clinical course of disease. Finally, this study also shows that those hospitalized LF infected patients with hypoalbuminemia and/or reversal of albumin-to-globulin ratio tend to have leucocytosis and experience prolonged duration of illness.

## Literature review on Lassa fever infection

Lassa fever (LF) infection is an acute viral hemorrhagic fever disease found mainly in Sub-Saharan West-Africa, including Nigeria [1]. Lassa mammarenavirus (LASV) is an arenavirus that causes LF infection in humans and other primates. LASV is an emerging virus and a selected agent, requiring Biosafety Level 4-equivalent containment. The multimammate rat – *Mastomys natalensis* [1,2], was demonstrated to be the main reservoir (primary host) of this zoonotic virus in Sub-Saharan West Africa, able to shed LASV in its urine and feces without exhibiting visible symptoms of infestation [1,2]. It is estimated that approximately 100,000–300,000 LF infections occur annually, with an estimated 5,000 deaths [1,2]. LF infection is associated with immunosuppression, vasculopathy and inflammation with multisystemic involvement, leading in some cases to hematologic, hepatic, renal, neurologic and cardiovascular damage. One of the features of inflammation is hypoalbuminemia. Inflammatory process’ increase capillary permeability with resultant escape of plasma albumin, leading to the expansion of the interstitial space and increasing the distribution volume of albumin [2]. Some conditions could lead to increased vascular permeability for cells and plasma solutes. These include critical illness, trauma, chronic disease, life events, multiple or isolated organ failure, and cancer [2]. A previous study reported an association between low serum albumin, infection and increased morbidity or mortality outcome among critically ill patients [3].

Another possible factor associated with some health conditions is the albumin-to-globulin ratio. The level of albumin and globulin in the human body partially represents the nutritional status and immune system function, respectively. Albumin-to-globulin ratio has been reported as a prognostic factor in various disease conditions, including cancers. Also, it has been reported as a predictor of prognostic outcome in infectious disease such as H1N1 influenza viral infection [4]. It has also been indicated that albumin-to-globulin ratio has significant prognostic effect on the survival in different solid tumor patients including both nonmetastatic and metastatic patients [5]. But presently, there is a paucity of data on the association between hypoalbuminemia, reversal of albumin-to-globulin ratio and morbidity outcome in LF infection.

Therefore, this study was conducted to assess the association of hypoalbuminemia, reversal of albumin-to-globulin ratio and morbidity outcome in hospitalized LF-infected patients in a dedicated treatment center at Owo Local Government Area of Ondo State, south-western Nigeria.

## Methodology

### Study site

The study was conducted in the Infection Control Unit of Federal Medical Center, Owo, Ondo State, Nigeria.

### Study design & population

This study was designed to determine the association between hypoalbuminemia, reversal of albumin-to-globulin ratio and morbidity outcome among confirmed LF-infected patients. The was a descriptive retrospective study that involved primary data analysis of the medical case record files of LF-infected patients that were being managed at the Infection Control Unit of Federal Medical Center, Owo, Ondo State, Nigeria. The medical case record files of 83 recruited participants who met the study’s inclusion criteria out of the 100 confirmed LF-infected patients being managed at the center over a 12-month period from November 2018 to October 2019 were enrolled and used for this study.

### Inclusion criteria

- All confirmed LF-infected patients that were managed at the Infection Control Unit of the hospital provided they signed an informed consent to participate in this study.
- All confirmed LF-infected patients who had their serum protein, albumin and globulin results assayed.

### Exclusion criteria

- Those confirmed LF-infected patients who did not sign their informed consent were deemed ineligible and were therefore excluded from this study.
- Those confirmed LF-infected patients with incomplete data results needed for this study.
- Suspected LF cases that turned-out to be negative to the Lassa PCR test.

### Study outcome

The primary outcome for this study involved monitoring these LF-infected patients for elevated white blood cell (WBC) count, need for hemodialysis, acute kidney injury (AKI) occurrence, duration of illness, pregnancy miscarriage, bleeding diathesis, encephalopathy, Lassa PCR test at discharge and survival outcome. While, there was no any secondary outcome for this study.

### Equipments, materials & parameter definition

The equipments and materials used were quantitative PCR test machine, Model Rotor-Gene Q, S/N RO516117, Qiagen Hilden, Germany, manufactured in Malaysia, is used to amplify the viral particles for diagnostic purposes. Biochemistry analyzer Piccolo, type: TS-100C, S/N: 01014316120781, made in Latvia, Biosan SIA, is used to analyze the various biochemical parameters such as electrolytes, urea, creatinine, bicarbonates, chloride, including parameters for liver function analysis such as serum albumin level and serum total protein level. Extraction centrifuge, Eppendorf AG, S/N 5418FL724392, designed and engineered in Germany, assembled in USA, is used to spin the tubes containing the patient samples and buffers for the purpose of easy extraction and concentration of viral particles and serum samples for biochemical analytes. Automated Hematology analyzer pocH-100i, S/N G5669, Sysmex Corporation, 03/2018 made in Japan, is used to analyze hematological parameters such as hemoglobin, hematocrit, red cell indices, WBCs and platelet count. Glove box, PLAS LABS, Model 815-PGB/EXP, S/N 14618, Manufactured in 5/11/18 at Lansing, MI 48906, is a level 3 biosafety device used in the process of sample decontamination. Other equipments include consumables, vacutainer sample collection tubes and overall wear of personal protective biosafety gowns.

Concerning parameter definition, the serum total protein and albumin values were gotten from analysis using Picolo machine, while serum globulin value was derived by subtracting serum albumin from serum total protein. The albumin-to-globulin ratio was calculated by dividing serum albumin level with serum globulin level. AKI was defined as increased serum creatinine >0.3 mg/dl (or >26.5 mmol/l) within 48 h or reduced urine output volume (<0.5 ml/kg/h for at least 6 h) according to the National Kidney Foundation-Kidney Disease Outcome Quality Initiative (NKF-KIDGO) criteria. We defined bleeding diathesis as bleeding from any orifice (epistaxis, oral mucosa, vagina, urethra or intravenous sites) with a crude clotting time more than 20 min. Prothrombin time and activated partial thromboplastin time with kaolin could not been done due to financial resource constrain.

### Data source

Records of admitted LF-infected patients were obtained from the Infection Control Unit of Federal Medical Center, Owo, Ondo State, Nigeria from November 2018 to October 2019.

### Data analysis

The statistical package software for social sciences (SPSS^R^) version 21.0 was used for data entry, validation and analysis. Frequencies and proportions were generated and presented using tables and figures where necessary. Bivariate analysis was carried out using Pearson correlation test, Chi-square test and Fisher exact test for comparison of proportions for categorical variables and independent student t-test for comparison of means for continuous variables where necessary. The level of statistical significance was defined by p < 0.05.

### Ethical consideration & informed consent

Ethical clearance/approval was obtained from the Institutional Health Research Ethical Committee before commencing this study. In addition, a duly signed written informed consent was obtained from each of the patients whose medical case record files were used while the medical case record files for those who did not sign their informed consent were excluded from this study. Participants’ confidentiality was respected and maintained by ensuring that no unauthorized person had access to the information on the data information sheets, that no information can be traced to the subjects (as coding system was used for the data information sheets instead of writing the patients’ names on them) and no unauthorized use of information was made.

## Results

Out of the 100 assessed records of confirmed LF-infected patients that were being managed at the center during the study period under review; only 83 recruited participants had complete records with a mean age of 33.95 ± 18.80 years and 47 (56.6%) were males. Comorbidities identified among these participants were hypertension in six (7.2%), diabetes mellitus in one (1.2%), hepatitis B viral infection in five (6.0%) and HIV infection in one (1.2%).

Table 1 reveals that out of the 83 patients with complete records, 66 (79.5%) had hypoalbuminemia. Furthermore, Table 1 shows statistically significant associations between serum albumin level versus total WBC count (p < 0.0001), AKI (p = 0.009), bleeding diathesis (p < 0.0001) and occurrence of pregnancy miscarriage (p < 0.0001). It is worthy to note that calculating the mean of the parameters for pregnant women who had miscarriage occurrence versus those who did not when we do not consider the whole 83 recruited participants that had complete records will result in statistical bias and this will be error prone because this was an observational study for which we reported our findings based on the number of pregnant patients during the study period to serve as a template for a larger cohort study.

**Table 1.** Association of hypoalbuminemia and health variables among Lassa fever-infected patients during admission.

Variable	Marked hypoalbuminemia (albumin <25 g/l) (%)	Mild hypoalbuminemia (albumin 25–34.99 g/l) (%)	Normal albumin levels (albumin 35–45 g/l) (%)	p-value
White blood cell count				
– Elevated (>11,000/mm^3^)	11 (57.9%)	6 (11.5%)	0 (0%)	<0.0001^†^
– Normal (4–11,000/mm^3^)	8 (42.1%)	27 (52.0%)	8 (66.7%)	
– Low (<4000/mm^3^)	0 (0%)	19 (36.5%)	4 (33.3%)	
Acute kidney injury				
– Yes	8 (40%)	7 (13.7%)	0 (0%)	0.009^†^
– No	12 (60%)	44 (86.3%)	12 (100%)	
Duration of illness (days)				
– 0–10	1 (5%)	3 (5.9%)	2 (16.7%)	0.329
– 11–20	7 (35%)	22 (43.1%)	7 (58.3%)	
– >20	12 (60%)	26 (51%)	3 (25%)	
Bleeding diathesis				
– Yes	12 (20.7%)	5 (38.5%)	0 (0%)	<0.0001^†^
– No	46 (79.3%)	8 (61.5%)	12 (100)	
Encephalopathy				
– Yes	4 (20%)	3 (5.9%)	0 (0%)	0.082
– No	16 (80%)	48 (94.1%)	12 (100%)	
Pregnancy miscarriage				
– Yes	3 (100%)	1 (50%)	0 (0%)	<0.0001^†^
– No	0 (0%)	1 (50%)	1 (100%)	
Patient outcome				
– Discharged	16 (80%)	47 (92.2%)	12 (100%)	0.269
– Died	4 (20%)	3 (5.9%)	0 (0%)	
– Transferred	0 (0%)	1 (1.9%)	0 (0%)	

† p < 0.05 is considered to be statistically significant.

Furthermore, Table 2 shows the distribution of albumin-to-globulin ratio among the LF-infected patients during admission with 74 (89.2%) had low albumin-to-globulin ratio, while six (7.2%) had normal albumin-to-globulin ratio, and three (3.6%) had high albumin-to-globulin ratio.

**Table 2.** Distribution of albumin-to-globulin ratio among the Lassa fever-infected patients during admission.

Reference category of albumin-to-globulin ratio	Frequency (n)	Percentage (%)
Low (≤0.90)	74	89.2
Normal (0.91–1.09)	6	7.2
High (≥1.10)	3	3.6
**Total**	83	100

Regarding Pearson correlation test, we observed that there was a statistically significant weak strength, negative correlation between serum albumin level and total WBC count (r = -0.366; p = 0.001) among the LF-infected patients in this study. Also, there was a statistically significant weak strength, negative correlation between serum albumin level and duration of illness (r = -0.274; p = 0.012) among the LF-infected patients in this study.

Furthermore, there was a statistically significant poor strength, negative correlation between albumin-to-globulin ratio and total WBC count (r = -0.244; p = 0.027) among the LF-infected patients in this study. In addition, there was a statistically significant weak strength, negative correlation between albumin-to-globulin ratio and duration of illness (r = -0.280; p = 0.010) among the LF-infected patients in this study.

## Discussion

As earlier reiterated, this study aimed to determine the association between hypoalbuminemia, reversal of albumin-to-globulin ratio and morbidity outcome among confirmed LF-infected patients.

Comorbidities as noticed in some of the patients in this study may worsen LF condition. However, a Nigerian study did not observe a significant association between comorbidities and mortality due to LF infection [6]. Although the study acknowledged that the poor capacity of healthcare facilities in resource-poor settings to appropriately diagnose and manage all underlying comorbidities may have masked the contribution of comorbidities to mortality.

In this study, there was a statistically significant association between serum albumin level and WBC count; this association is known to be associated with inflammation [2]. Findings from a study in Irrua, Nigeria, showed that there was a higher mean value of total WBC count among LF-infected patients who died compared with those who survived with statistical significance [7]. Also, the study in Irrua also noted lower serum albumin levels among the patients that died compared with survivors and the difference was statistically significant. Similar study in the USA of America identified low albumin levels and high white blood counts as both biomarkers of inflammation [8]. These biomarkers were found to predict the future risk of chronic kidney disease.

This study showed that all LF-infected patients who had AKI had some form of hypoalbuminemia. It also identified a statistically significant association between serum albumin level and AKI. AKI is a frequent complication of LF infection that is associated with poor outcome. In Irrua, it was found that among the complications of LF infection, AKI has the highest overall incidence of up to one-fourth of hospitalized LF-infected patients with case fatality rate as high as 60% [7]. AKI may arise as a result of prerenal insult, renal tubular injury and/or interstitial nephritis [9]. The occurrence of AKI may also predict the future risk of chronic kidney disease in these categories of LF-infected patients.

Among the six pregnant women in this study, significant hypoalbuminemia was recorded for four who had miscarriage as against the other two with a statistically significant level (p < 0.001). Hypoalbuminemia was found to be an independent predictor of negative pregnancy outcome among patients with systemic lupus erythematosus in a study done at China [10].

Pearson correlation test revealed that there were statistically significant weak strength, negative correlations between serum albumin level versus total WBC count (r = -0.366; p = 0.001) and duration of illness (r = -0.274; p = 0.012) among the LF-infected patients in this study. This implies that those hospitalized LF-infected patients with hypoalbuminemia tend to have leucocytosis and experience prolonged duration of illness in this study.

Other possible biomarker of LF infection is the reversal of albumin-to-globulin ratio which can provide healthcare professionals with a clue as to the cause of the change in protein levels. Majority of the LF-infected patients in this study had low albumin-to-globulin ratio. This ratio has been used as prognostic factor in different conditions [4–11]. It has been shown to predict morbidity outcomes of H1N1 infection [4]. Studies have also identified the prognostic role of albumin-to-globulin ratio in predicting clinical outcomes and survival in cancers [5,11].

Also, Pearson correlation test revealed that there was a statistically significant poor strength, negative correlation between albumin-to-globulin ratio versus total WBC count (r = -0.244; p = 0.027) among the LF-infected patients in this study. While at the same time, there was a statistically significant weak strength, negative correlation between albumin-to-globulin ratio versus duration of illness (r = -0.280; p = 0.010) among the LF-infected patients in this study. This implies that those hospitalized LF-infected patients with low or reversal of albumin-to-globulin ratio tend to have leucocytosis and experience prolonged duration of illness in this study.

The strength with limitation of this particular study was that the findings observed from this research are only exclusively applicable to LF-infected patients in clinical practice settings. In addition, we would have desired to use a larger sample size for this cohort study; but because of the restricted local endemic nature of LF infection in Nigeria, only 83 recruited participants with complete data records were enrolled for the period under consideration.

## Conclusion

There is a baseline hypoalbuminemia and reversal of albumin-globulin ratio among confirmed LF-infected patients. Based on these findings, the level of albumin and albumin-to-globulin ratio at presentation may serve as simple early biomarkers to identify patients at high risk for a complicated clinical course of disease. Finally, this study also shows that those hospitalized LF-infected patients with hypoalbuminemia and/or reversal of albumin-to-globulin ratio tend to have leucocytosis and experience prolonged duration of illness.

## Future perspective

Future research study is required using a relatively large sample size number of hospitalized LF-infected patients and a multicenter study; as we plan to carry out a research survey to ascertain the nutritional status and immunologic function of people living in Lassa viral infection endemic communities and to determine the association/correlation between albumin, protein malnutrition disorder, LF infection and prognostic outcomes.

Also, we plan to conduct a prospective analytical interventional longitudinal study with salt-poor albumin solution among experimental cohort group of hospitalized LF-infected patients with baseline hypoalbuminemia at presentation that has experienced interventional treatment with salt-poor albumin; and then monitor their morbidity or treatment-survival outcome in comparison with both control cohort group of hospitalized LF-infected patients with baseline hypoalbuminemia at presentation that has no any interventional treatment with salt-poor albumin and control cohort group of hospitalized LF-infected patients with baseline normoalbuminemia at presentation.

In addition, we plan to conduct a prospective description study among cohort group of hospitalized LF-infected pregnant patients with respect to miscarriage occurrence; since the number enrolled in this present study was very scanty, this could be error prone and lead to statistical bias if the mean of parameters for these hospitalized LF-infected pregnant women were calculated without considering the whole sample size of 83 recruited hospitalized LF-infected study participants that had completed records.

**Executive summary**

- As of this present moment, there is paucity of data on the association between hypoalbuminemia, reversal of albumin-to-globulin ratio and morbidity outcome in Lassa fever (LF) infection; while this study serves to bridge this scientific gap.
- A higher mean value of total white blood cell count was observed among confirmed LF-infected patients with hypoalbuminemia (p < 0.0001) and reversal of albumin-to-globulin ratio (p <0.0001) when compared to patients with normal values, respectively. Also, this study showed statistically significant associations between serum albumin level versus total white blood cell count (p < 0.0001), acute kidney injury (p = 0.009), bleeding diathesis (p < 0.0001) and occurrence of pregnancy miscarriage (p < 0.0001).
- There is a baseline hypoalbuminemia and reversal of albumin-to-globulin ratio among confirmed LF-infected patients. Based on these findings, the level of albumin and albumin-to-globulin ratio at presentation may serve as simple early biomarkers to identify patients at high risk for a complicated clinical course of disease.
- This study also shows that those hospitalized LF-infected patients with hypoalbuminemia and/or reversal of albumin-to-globulin ratio tend to have leucocytosis and experience prolonged duration of illness.
